# Non-O1/non-O139 *Vibrio cholerae* septicaemia in a Saudi man: a case report

**DOI:** 10.1099/jmmcr.0.005077

**Published:** 2017-02-28

**Authors:** Reham Kaki, Dalia El-Hossary, Asif Jiman-Fatani, Rahaf Al-Ghamdi

**Affiliations:** ^1^​ Department of Medicine, Faculty of Medicine, King Abdulaziz University, Jeddah, Saudi Arabia; ^2^​ Department of Infection Control and Environmental Health, King Abdulaziz University Hospital, Jeddah, Saudi Arabia; ^3^​ Clinical and Molecular Microbiology Laboratory, King Abdulaziz University Hospital, Jeddah, Saudi Arabia; ^4^​ Department of Medical Microbiology and Immunology, Faculty of Medicine, Zagazig University, Zagazig, Egypt; ^5^​ Department of Medical Microbiology and Parasitology, Faculty of Medicine, King Abdulaziz University, Jeddah, Saudi Arabia

**Keywords:** Non-O1/Non-O139 *Vibrio cholerae*, septicaemia

## Abstract

**Background.** The non-O1/non-O139 serogroups of *Vibrio cholerae* occur in diverse natural niches, and usually cause mild and self-limiting gastrointestinal illness. However, they have well-documented potential to cause invasive and extra-intestinal infections among immunocompromised patients. Furthermore, their ability to grow in low-salinity surface water, and the existence of asymptomatic human carriers, suggest novel acquisition routes for this unusual infection, even in people without obvious risk factors.

**Case presentation.** A 62-year-old man presented with epigastric pain, vomiting and fever. The patient had a history of diabetes and cholecystectomy, although our initial examination did not reveal any significant findings that might indicate *V. cholerae* infection. However, blood cultures subsequently revealed the presence of *V. cholerae*, which was positively identified using both conventional and modern non-conventional technologies. The identity of the *V. cholerae* isolate was confirmed using Vitek MS (matrix–assisted laser desorption ionization-time of flight MS) and the FilmArray system, in addition to its initial identification using the Vitek 2 system. The septicaemia was successfully treated using a 14 day course of ciprofloxacin.

**Conclusion.** The present case highlights the need to remain highly suspicious of non-O1/non-O139 *V. cholerae* infections in patients with known risk factors, as well as in healthy individuals with epidemiological exposure and compatible clinical symptoms. Special care should be taken to avoid false-positive results from confirmatory laboratory tests, as the organism can grow in fresh water, and the results should be verified using multiple methods.

## Abbreviation

MALDI-TOF, matrix-assisted laser desorption/ionization time-of-flight.

## Introduction

*Vibrio cholerae* is a Gram-negative motile facultative anaerobic bacterium that is usually found in municipal water and estuarine environments [1–3]. This bacterium causes approximately 1.4–4.3 million cases of acute diarrhoeal illness and 28 000–142 000 deaths worldwide each year [4]. Furthermore, this bacterium has >200 serogroups, based on the lipopolysaccharide surface O-antigen, with the O1 and O139 serogroups causing most outbreaks of epidemic and pandemic cholera [5, 6]. Although the non-O1/non-O139 serogroups generally cause mild and transient gastrointestinal illness, wound infections or ear infections among healthy individuals, they can also cause severe wound infections or sepsis, and are associated with high mortality rates among immunocompromised individuals [7, 8]. Non-O1/non-O139 strains are also notable for their ability to grow in low-salinity conditions [9]. Non-O1 *V. cholerae* infections are thought to be under-detected and under-reported, because of a low level of awareness among clinicians and the non-use of standard culture media [such as thiosulfate-citrate-bile salts-sucrose (TCBS) agar] for isolation of the organism [10]. Furthermore, the O1 and O139 strains can produce cholera toxin and toxin-coregulated pilus, which cause secretory diarrhoea and intestinal colonization, respectively [5, 11–13]. Although non-O1 strains generally lack these two toxins, they can carry other virulence genes, such as genes encoding the regulatory ToxR protein and the *Vibrio parahaemolyticus* type three secretion system. Non-O1 strains also produce heat stable enterotoxin, α-haemolysin, repeats-in-toxin and type 3 secretion systems [14, 15].

## Case report

A 62-year-old Saudi man presented to our tertiary teaching hospital (King Abdulaziz University Hospital) with epigastric pain, intermittent vomiting and a 3 day fever. However, he did not have a cough, diarrhoea or dysuria, and denied travel during the past year, seafood consumption or swimming in brackish water. He had diabetes for the past 20 years, but maintained good glycaemic control using regular insulin therapy. Three years before his presentation, he had undergone cholecystectomy for chronic calculous cholecystitis.

Our examination revealed that the patient had a temperature of 38.5 °C, a blood pressure of 120/60 mmHg (16/8 kPa), a heart rate of 110 beats min^−1^ and a respiratory rate of 20 breaths min. He was alert and oriented, and exhibited no signs of chronic liver disease. There was mild-to-moderate epigastric tenderness, although no guarding or rigidity. He did not have focal neurological signs, nuchal rigidity or peripheral lymphadenopathy.

## Investigations

Two sets of blood samples were drawn for culture at admission and before any antimicrobial treatment. The blood cultures were performed using an automated blood culture system (BacT/ALERT 3D; Organon Teknika). Ten millilitres of blood were inoculated into a set of aerobic and anaerobic blood culture bottles, which were loaded into the BacT/ALERT 3D system, and incubated for a maximum of 5 days. A complete blood count revealed haematocrit levels of 42.1 %, a white blood cell count of 15 700 cells mm^−3^ (89 % polymorphonuclear cells, 3.7 % lymphocytes and 6.9 % monocytes) and a platelet count of 141 000 platelets mm^−3^. Liver function tests revealed total bilirubin levels of 14 µmol l^−1^, alkaline phosphatase levels of 82 U dl^−1^, aspartate transaminase levels of 49 U dl^−1^, alanine transaminase levels of 81 U dl^−1^ and albumin levels of 32 g l^−1^. Serological tests revealed no evidence of infection with hepatitis A/B/C or human immunodeficiency virus.

**Fig. 1.**
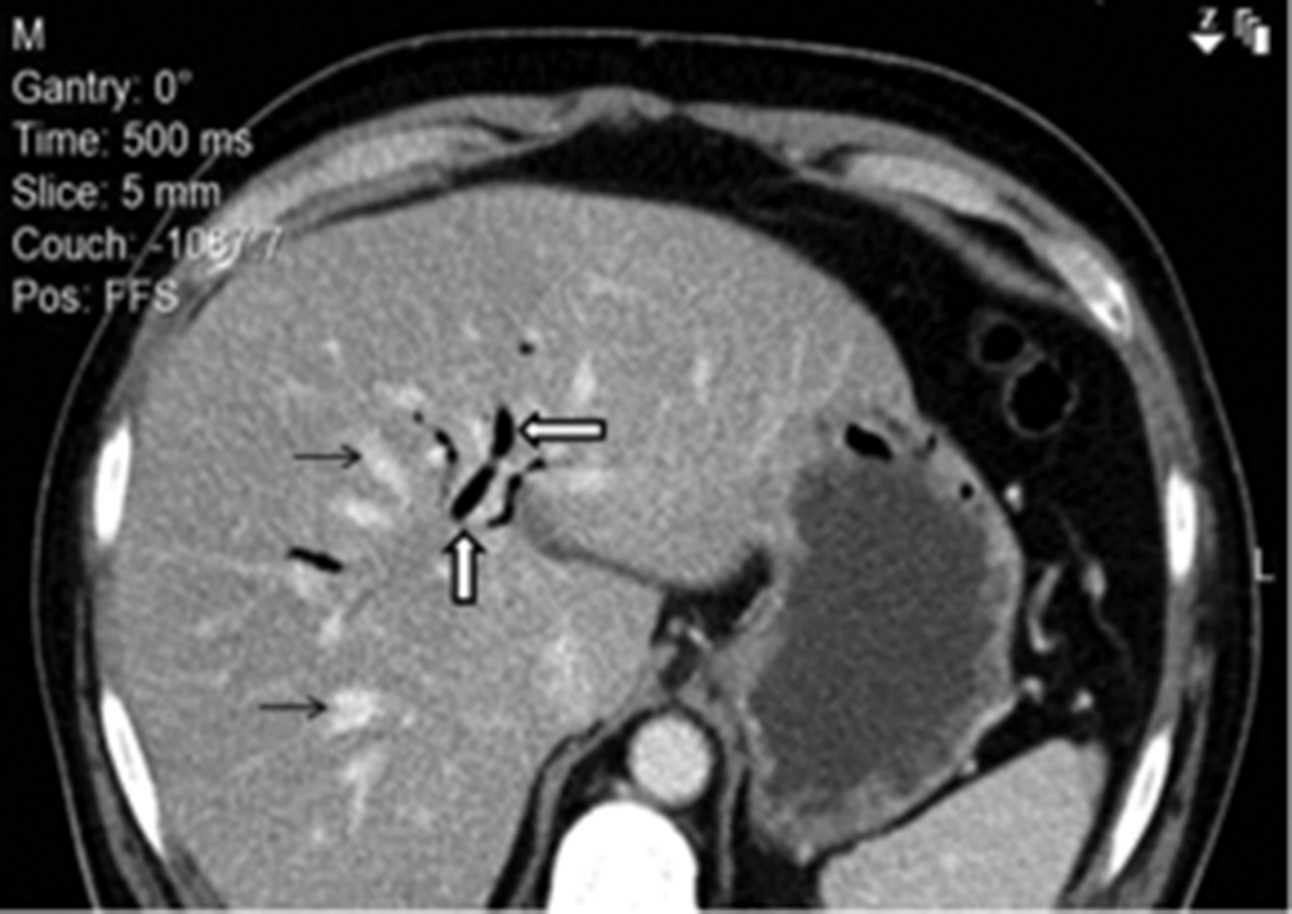
Axial contrast-enhanced computed tomography image of the patient’s abdomen revealing pneumobilia (thick white arrows) and dilated intrahepatic biliary radicles (thin black arrows).

Chest radiography did not reveal any air under the diaphragm, and his lung parenchyma and cardiomediastinal silhouette appeared to be normal. Ultrasonography of the abdomen did not reveal any stones or dilation of the common bile duct. Contrast-enhanced computed tomography of the abdomen at admission revealed dilated intra-hepatic biliary radicles with periampullary thickening and pneumobilia (Fig. 1). No features of pancreatitis were observed. For the pneumobilia, the imaging results were reviewed by a radiologist, and additional testing was performed, which included esophagogastroduodenoscopy. However, these tests ruled out biliary-enteric anastomosis, hepatopancreatic sphincter incompetence and spontaneous biliary-enteric fistula.

## Diagnosis

The blood culture bottles revealed microbial growth after approximately 11 h of incubation. Smears were prepared from the blood culture bottles, and were evaluated using Gram's stain. The positive blood culture bottles were subsequently sub-cultured onto blood agar, MacConkey agar and chocolate agar plates (Saudi Prepared Media Laboratories), which were incubated for 18–24 h.

**Fig. 2.**
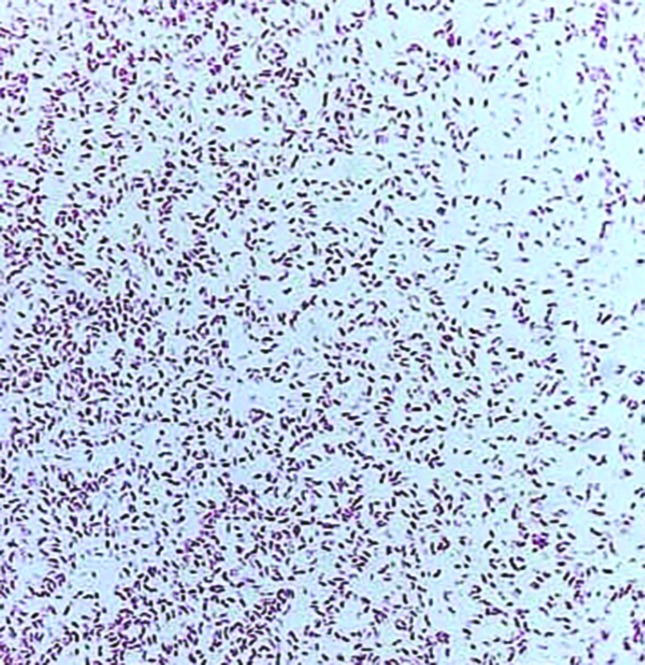
Gram-negative curved bacilli (*V. cholera*) from a culture plate. Magnification ×100.

The stained smears revealed the presence of comma-shaped Gram-negative bacilli. Further identification and antimicrobial-susceptibility testing were performed using the VITEK 2 system (bioMérieux) according to the manufacturer’s instructions. The organism was identified as *V. cholerae*, with a 99 % probability, although there were no results for the susceptibility testing, as *V. cholerae* is not included in the antimicrobial-susceptibility panels for the VITEK 2 system. The results were verified via subculture on TCBS medium, which yielded characteristic yellow colonies (Fig. 2). We subsequently re-cultured a colony from the TCBS culture, and confirmed that the organism was *V. cholerae*, with a 98 % probability, using the VITEK 2 system. Analysis of the growth on the TCBS medium using a VITEK matrix–assisted laser desorption ionization-time of flight (MALDI-TOF) mass spectrometer (bioMérieux) also confirmed the isolate as *V. cholerae*, with a probability of 96 %. The identity of the isolate was further verified using a FilmArray system (BioFire, bioMérieux), according to the manufacturer’s instructions.

A commercial slide agglutination test (MAST Diagnostics) was used to serotype the *V. cholerae* isolate, according to the manufacturer’s instructions. In brief, 1–2 colonies were emulsified in two 10 µl drops sterile 0.85 % saline solution on a clean glass slide. One drop (40 µl) O1 antiserum (MAST ASSURE *V. cholerae* polyvalent antisera) was added onto one drop of the emulsification, a similar amount of saline was added to the second drop (the control sample), and both drops were mixed. Similar procedures were performed using O139 antiserum. These tests revealed that the isolate was non-O1/non-O139 *V. cholerae*, as it did not react with the antisera. Given that our patient did not report a history of travel to an area with endemic cholera, eating raw shellfish or diarrhoea, we considered it likely that he was infected with a non-O1/non-O139 strain of environmental origin. Although these strains can cause serious disease in susceptible hosts, they do not represent a public-health threat (unlike toxigenic O1/O139 *V. cholerae*), and we did not seek to detect any virulence factors or toxigenicity.

The antimicrobial susceptibility of the *V. cholerae* isolate was tested using a standard disc diffusion method with ampicillin, piperacillin/tazobactam, ceftriaxone, gentamicin, ciprofloxacin, tetracycline, and trimethoprim/sulfamethoxazole. The zones of inhibition for the antibiotics were interpreted according to the 2015 Clinical and Laboratory Standards Institute guidelines. The results from this test revealed that the *V. choleraeisolate* was susceptible to piperacillin/tazobactam, trimethoprim/sulfamethoxazole, gentamicin, ceftriaxone and ciprofloxacin. Furthermore, the isolate was not resistant to any of the antimicrobial drugs that we tested.

Our search for the source of the bacteraemia included an assessment of biliary tumour markers. The patient’s carbohydrate antigen 19–9 levels were slightly elevated (49 IU ml^−1^; normal range 0–39 IU ml^−1^), although we found normal levels of α-fetoprotein (1.38 IU ml^−1^; normal range 0–5.8 IU ml^−1^) and carcinoembryonic antigen (2.46 ng ml^−1^; normal range 0–3.4 ng ml^−1^).

## Treatment

Based on the susceptibility-test results, the patient initially received intravenous piperacillin/tazobactam (4.5 g every 6 h). A stool culture was ordered at admission, although the sample was collected after the patient had received two doses of piperacillin/tazobactam, because the patient did not experience a bowel movement and the antibiotic treatment could not be delayed due to his clinical status. The antimicrobial therapy was subsequently changed to a 14 day course of ciprofloxacin (500 mg orally twice per day). Blood cultures that were performed 2 days after the completion of treatment were negative, although the patient required additional doses of insulin to maintain euglycaemia, which likely reflects the effects of the septicaemia. In addition, the patient’s fever and abdominal symptoms resolved, and his white blood cell counts normalized 2 days after the antimicrobial treatment. After an initial increase (which was likely due to the septicaemia), his liver enzyme levels also normalized.

## Outcome and follow-up

The patient was subsequently discharged and remained healthy during his follow-up visits at 1 and 3 months.

## Discussion

In this report, we describe a case of non-O1/non-O139 *V. cholerae* infection in an apparently healthy 62-year-old man. Despite a 20 year history of diabetes mellitus, he had normal blood glucose and glycated haemoglobin levels, and did not exhibit any evidence of compromised immunity. This case was remarkable for its negative history of travel, seafood intake and/or contact with brackish water, as well as the absence of gastrointestinal symptoms, other than epigastric discomfort and intermittent vomiting.

Zafari *et al.* [16] have reported asymptomatic carriage and prolonged faecal shedding of non-O1 *V. cholerae* among Iranian pilgrims who were returning from Saudi Arabia, which is an area that is endemic for the organism. Reports have also indicated that chronic biliary carriage of this organism can occur in patients who do not have diarrhoea [17, 18]. In contrast, Eltahawy *et al.* [19] have reported fatal septicaemia because of non-O1 *V. cholerae* in a patient with liver cirrhosis. This diagnosis was confirmed through the isolation of non-O1/non-O139 *V. cholerae* from two blood culture bottles, and verification of its morphological and biochemical characteristics. Interestingly, Lai *et al.* [20] have reported the isolation of non-O1 *V. cholerae* from the respiratory tract, bile, uterus, urine and cerebrospinal fluid. However, the present case only exhibited positive blood cultures (no positive urine or stool cultures) before the start of the antimicrobial treatment. Furthermore, it is likely that our findings were not related to laboratory contamination, as the skin was disinfected before the venepuncture, and both blood culture bottles yielded exclusive growth of *V. cholerae*, without any other skin commensals.

Luo *et al.* [21] have reported that non-O1 *V. cholerae* account for 1–3.4 % of all cases of acute watery diarrhoea, and a recent meta-analysis by Deshayes *et al.* [22] identified 350 reports of bacteraemia from non-O1/O139 *V. cholerae* in the MEDLINE database between 1974 and 2014. The authors observed that most cases involved middle-aged men (mean age 56 years), and that cirrhosis, malignancies, alcoholism, liver diseases, diabetes and iatrogenic exposures increased the risk of bacteraemia. Therefore, the present patient’s cholecystectomy, along with his long history of diabetes and probable intrahepatic/biliary pathology, might have increased his risk of non-O1 *V. cholerae* infection.

Pneumobilia is defined as the presence of air within the biliary tree of the liver. It indicates an abnormal communication between the biliary tract and the gastrointestinal tract such as the intestine, or infection by gas-forming bacteria. Most commonly, pneumobilia is associated with a biliary-enteric surgical anastomosis, an incompetent sphincter of Oddi or a spontaneous biliary-entericfistula [23–26].

Air can accumulate within the biliary system, secondary to reflux through the biliary sphincter. In our case, the reflux happened from iatrogenic incompetence of the sphincter after the patient's previous Endoscopic retrograde cholangiopancreatography (ERCP) and cholecystectomy 3 years previously, likely from sphincterotomy to allow stone removal [27, 28]. The patient improved after treatment with antibiotics, and the pneumobilia was thought to be an incidental finding secondary to the sphincterotomy performed during the ERCP.

Non-O1 *V. cholerae* infection is strongly associated with the consumption of seafood, such as oyster, fish, shrimp, clam, mussel and apple snail. The most common clinical symptoms include hypothermia or hyperthermia, diarrhoea (generally watery, although bloody and mucoid patterns are possible) and abdominal pain. Other presentations have included hepatic, prostate, cerebral and peritoneal abscesses, as well as pyomyositis, pneumonia, peritonitis, skin infection, cellulitis, necrotizing fasciitis, endophthalmitis, cholecystitis and meningitis [7, 29–37]. Hypotension, confusion and coma are associated with a poor prognosis, although gastrointestinal surgery provides an improved prognosis. However, death has occurred in approximately 33 % of the patients with non-O1 *V. cholerae* bacteraemia [22]. During the infection, the bacteria may enter the bloodstream from the intestine through the portal vein or the lymphatic system, although approximately 75 % of all cases have no history of aquatic exposure or seafood consumption, which suggests that there are alternate routes for bacterial entry into the bloodstream [22]. Reports have also indicated the possibility of asymptomatic human carriers, and carriage in wild and domestic animals [38–40]. Therefore, these factors likely contributed to the present case, as the patient denied having a history of travel, seafood consumption and/or exposure to brackish water.

Unlike classical cholera, antimicrobial treatment is essential for the management of extra-intestinal *V. cholerae* infections. However, antimicrobial-susceptibility testing is critical, given the absence of standard therapeutic guidelines for these infections. Various reports have indicated that non-O1/non-O139 *V. cholerae* isolates are susceptible to β-lactams, fluoroquinolones, trimethoprim/sulfamethoxazole, tetracycline and chloramphenicol [41]. Nevertheless, a study by Datta *et al.* [42] revealed high rates of resistance among non-O1/non-O139 *V. cholerae* isolates to nalidixic acid, ampicillin, furazolidone and streptomycin, although the isolates were generally susceptible to gentamicin (96 %), tetracycline (80 %) and chloramphenicol (80.4 %). The isolates from the present case were susceptible to piperacillin/tazobactam, co-trimoxazole, aminoglycoside, third-generation cephalosporins and fluoroquinolones, and the patient quickly improved after a 14 day course of ciprofloxacin.

Similar case reports have been published by many authors throughout the world [43], although the novel aspect of the present case is our use of modern non-conventional diagnostic technologies (i.e. the VITEK MALDI-TOF MS and FilmArray systems). The VITEK MALDI-TOF MS system provides a rapid method for identifying bacteria and fungi from microbial cultures, and the results from the present case indicated *V. cholerae* with a 96 % probability. The FilmArray system performs automated nested multiplex PCR and high-resolution melting analysis to detect and identify multiple nucleic acid targets from clinical specimens. The sample is loaded into a FilmArray pouch, the pouch is placed on the instrument, and the Film Array system automatically extracts nucleic acids from the sample and amplifies the pathogen-specific DNA sequences that are targeted by the assays. The resulting PCR products are evaluated using DNA melting analysis and the results are automatically interpreted and presented by the FilmArray software in an easy-to-understand report. The FilmArray results from the present case were also positive for *V. cholerae*. Furthermore, we used conventional laboratory diagnostic methods, such as biochemical reactions and identification using the VITEK 2 system (bioMérieux), which indicated *V. cholerae* with 98 % probability. To our knowledge, this is the first case that has involved both conventional and modern non-conventional methods to identify a *V. cholerae* strain.

### Conclusions

The present case highlights the need to remain highly suspicious of non-O1/non-O139 *V. cholerae* infections in patients with known risk factors, as well as in healthy individuals with epidemiological exposure and compatible clinical symptoms. Special care should be taken to avoid false-positive results from confirmatory laboratory tests, as the organism can grow in fresh water, and the results should be verified using multiple methods. Furthermore, future research should be directed towards understanding the prevalence and effects of non-O1/non-O139 *V. cholerae* infection and its associated illnesses in Saudi Arabia, in order to reduce the related morbidity and mortality rates.
